# P-1650. Temporal Trends in Serious Adverse Events Associated with Oral Antivirals During the COVID-19 Pandemic: Insights from the FAERS Database (2020–2023)

**DOI:** 10.1093/ofid/ofaf695.1825

**Published:** 2026-01-11

**Authors:** Ashin Siby, Manu Mathew, Jose T John

**Affiliations:** Durdans Hospital, Colombo, Western Province, Sri Lanka; Durdans Hospital, Colombo, Western Province, Sri Lanka; Durdans Hospital, Colombo, Western Province, Sri Lanka

## Abstract

**Background:**

The COVID-19 pandemic led to the widespread use of emergency-authorized and repurposed oral antivirals, including remdesivir (initial IV use, later oral analogues), favipiravir, molnupiravir, and nirmatrelvir-ritonavir (Paxlovid). While these agents have played a key role in outpatient COVID-19 management, their real-world safety profiles remain under continuous evaluation. This study aimed to assess the temporal trends and disproportionality of serious adverse events (SAEs) associated with oral antivirals during the pandemic using the U.S. FDA Adverse Event Reporting System (FAERS).

**Figure:**
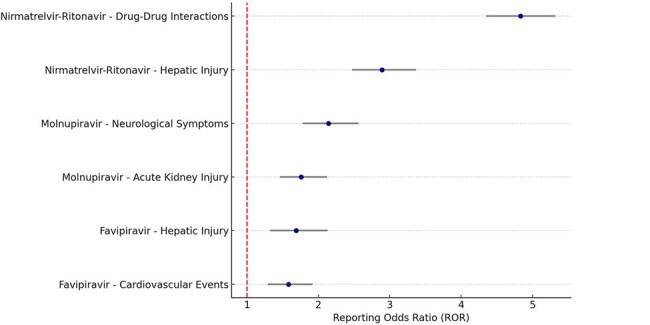
Forest Plot: Serious Adverse Events from Oral Antivirals (FAERS 2020–2023) This forest plot presents Reporting Odds Ratios (RORs) and 95% confidence intervals for serious adverse events linked to oral antivirals. Nirmatrelvir-ritonavir showed the strongest signals for drug–drug interactions (ROR: 4.83) and hepatic injury (ROR: 2.89). Molnupiravir was associated with neurological events and AKI. These findings highlight real-world safety patterns that inform risk-benefit assessment during antiviral therapy.

**Methods:**

A retrospective analysis of FAERS data from January 2020 to December 2023 was conducted. Reports involving favipiravir, molnupiravir, and nirmatrelvir-ritonavir were extracted. SAEs were defined per FDA criteria (death, life-threatening event, hospitalization, disability). Event categories included hepatic injury, acute kidney injury (AKI), cardiovascular events, hypersensitivity, and drug–drug interactions. Disproportionality was assessed using Reporting Odds Ratios (ROR) with 95% confidence intervals (CI). Annual report volume and severity trends were also analyzed.

**Results:**

A total of 11,547 SAE reports related to oral antivirals were included. Nirmatrelvir-ritonavir accounted for the majority (62.5%), with significant signals for drug–drug interactions (ROR: 4.83, 95% CI: 4.35–5.32) and hepatic injury (ROR: 2.89). Molnupiravir was associated with neurological symptoms (ROR: 2.14) and AKI (ROR: 1.76). Reports peaked in mid-2022 and declined with reduced community transmission and updated prescribing guidelines.

**Conclusion:**

This FAERS-based analysis highlights evolving safety trends of oral antivirals during the COVID-19 pandemic. Nirmatrelvir-ritonavir demonstrated notable risks for hepatotoxicity and interaction-related events, emphasizing the importance of medication reconciliation and patient monitoring. Real-time pharmacovigilance remains critical for safe deployment of emerging antivirals in outpatient care.

**Disclosures:**

All Authors: No reported disclosures

